# Supplementary material for: Effects of Spent Substrate of Oyster Mushroom (Pleurotus ostreatus) on Feed Utilization and Liver Serum Indices of Hu Sheep from the Perspective of Duodenal Microorganisms
Source: Animals (Basel). 2024 Nov 26;14(23):3416. doi: 10.3390/ani14233416 (PMC11639990; doi:10.3390/ani14233416)
Supplement: Supplementary file 1 [file animals-14-03416-s001.zip › animals-3294536-supplementary.pdf]

Supplementary Material

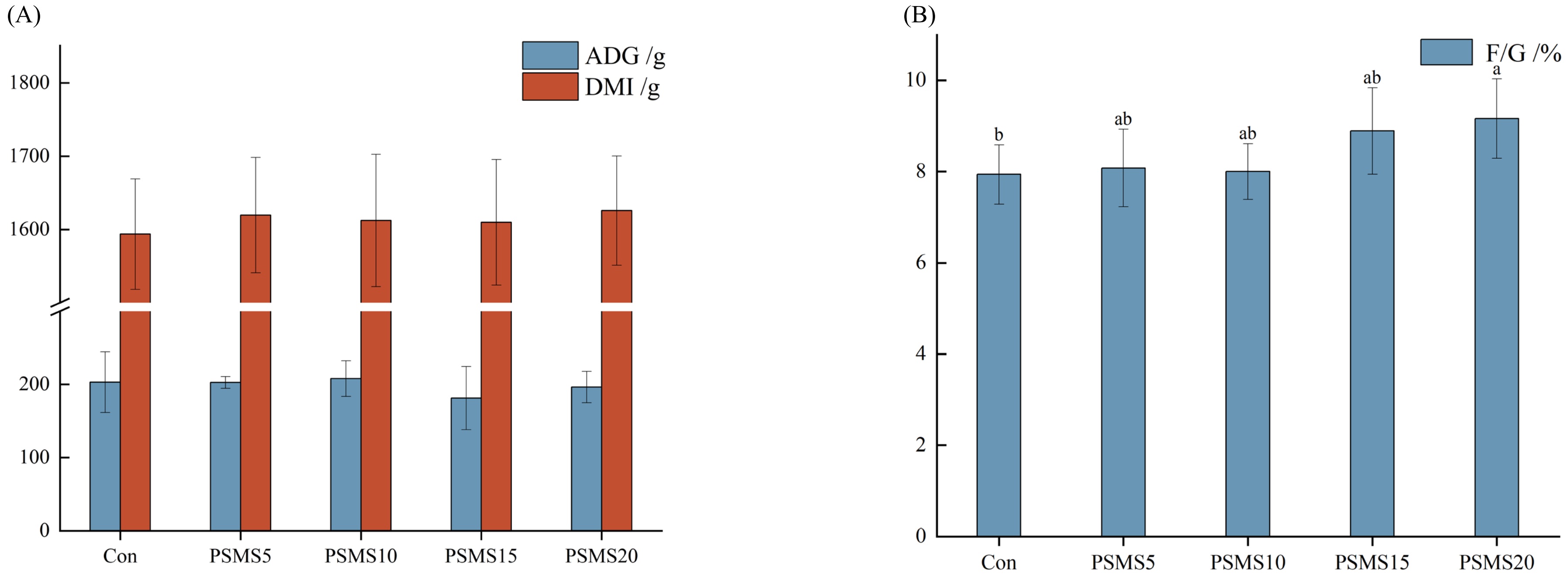

Supplementary figure S1. Feed utilization of sheep in five groups. (A). Average daily gain (ADG) and Dry matter intake (DMI) of five groups; (B). Feed-to-gain ratio (F/G) of five groups. The *P*-values for ADG, DMI, and F/G are 0.475, 0.457, and 0.045, respectively.

Supplementary Table S1. Statistical Analysis of Sample Sequencing Data Processing Results

Sample ID	16S*					ITS*				
	Raw CCS	Clean CCS	Effective CCS	Average length(bp)	Effective(%)	Raw CCS	Clean CCS	Effective CCS	Average length(bp)	Effective(%)
A1	6675	6620	6577	1452	98.53	8224	8224	8184	582	99.51
A2	8157	8117	8010	1455	98.20	7504	7502	7468	598	99.52
A3	8152	8114	8064	1461	98.92	7498	7494	7491	619	99.91
A4	8010	7989	7946	1460	99.20	7042	7037	6983	625	99.16
A5	7901	7838	7762	1455	98.24	7133	7133	7121	593	99.83
B1	8445	8419	8304	1461	98.33	7685	7685	7685	644	100.00
B2	6968	6966	6919	1463	99.30	7698	7697	7648	622	99.35
B3	8181	8081	8017	1451	98.00	8385	8385	8375	612	99.88
B4	7616	7424	7389	1448	97.02	7292	7290	7276	601	99.78
B5	8458	8430	8349	1454	98.71	6291	6290	6288	601	99.95
C1	7997	7971	7848	1457	98.14	7125	7125	7076	604	99.31
C2	7250	7228	7142	1455	98.51	7855	7854	7851	616	99.95
C3	6648	6616	6561	1458	98.69	7473	7473	7465	654	99.89
C4	7811	7768	7651	1455	97.95	7540	7539	7533	620	99.91
C5	7817	7770	7727	1455	98.85	6580	6577	6574	595	99.91
D1	6770	6755	6627	1461	97.89	8054	8054	8049	623	99.94
D2	6816	6777	6689	1459	98.14	7633	7633	7604	622	99.62
D3	7040	6990	6815	1460	96.80	7923	7920	7917	615	99.92
D4	7692	7678	7660	1460	99.58	7531	7529	7515	637	99.79
D5	8340	8274	8222	1456	98.59	5284	5282	5250	619	99.36
E1	5647	5622	5587	1460	98.94	8068	8068	8051	620	99.79
E2	8531	8510	8446	1458	99.00	8307	8305	8298	603	99.89
E3	8239	8228	8149	1458	98.91	7180	7179	7161	603	99.74
E4	6652	6642	6479	1461	97.40	7515	7513	7509	609	99.92
E5	7042	6992	6944	1453	98.61	7322	7321	7302	610	99.73

* Raw-CCS: Counts of identified CCS reads in the sample; Clean CCS: Counts of clean CCS reads (post primer removal and length filtration); Effective-CCS: Counts of effective CCS reads after chimeric reads removal; Average length(bp): Average length reads in the sample; Effective(%): Percentage of effective CCS reads in raw reads.

Supplementary Table S2. Biological classification of bacterial and fungal samples

Sample ID	16S							ITS						
	Kindom	Phylum	Class	Order	Family	Genus	Species	Kindom	Phylum	Class	Order	Family	Genus	Species
A1	1	13	20	39	64	108	125	1	6	13	17	24	34	45
A2	1	15	22	43	68	111	159	1	9	22	37	47	64	80
A3	1	14	21	41	70	123	171	1	5	12	16	21	24	26
A4	1	12	20	50	83	148	219	1	10	24	43	70	95	106
A5	1	17	24	48	85	143	214	1	8	18	25	37	46	56
B1	1	14	22	38	63	111	133	1	3	8	12	18	27	34
B2	1	11	19	41	66	123	181	1	8	13	21	30	41	60
B3	1	13	20	41	74	119	165	1	9	17	24	35	46	61
B4	1	12	19	41	67	118	153	1	6	11	13	21	26	33
B5	1	14	22	42	71	121	175	1	5	12	16	25	27	37
C1	1	14	24	56	95	161	231	1	7	16	20	29	40	53
C2	1	20	31	57	92	163	213	1	7	13	19	23	24	32
C3	1	13	20	42	76	138	184	1	8	14	19	23	30	36
C4	1	12	21	40	67	112	163	1	6	10	12	15	18	23
C5	1	12	18	44	75	138	192	1	4	10	14	17	17	24
D1	1	13	20	40	66	121	178	1	7	12	14	19	21	36
D2	1	13	21	47	78	151	232	1	8	14	20	27	33	43
D3	1	13	20	40	65	117	180	1	6	14	19	23	26	36
D4	1	15	23	47	81	140	207	1	8	16	21	34	37	51
D5	1	14	22	47	82	135	173	1	8	15	19	31	38	45
E1	1	14	23	49	90	140	216	1	6	13	20	30	37	53
E2	1	15	24	50	83	157	217	1	6	15	19	29	31	40
E3	1	15	25	55	95	161	221	1	3	7	9	14	16	24
E4	1	17	23	51	90	161	229	1	5	12	15	25	27	38
E5	1	15	25	44	77	132	189	1	6	13	17	26	32	37
Total	1	23	37	86	162	327	502	1	10	32	70	117	189	263

Supplementary Table S3. Composition of duodenum bacteria and fungus at the phylum level %

Composition	Groups†					*P*-value
	Con	PSMS5	PSMS10	PSMS15	PSMS20	
**Bacteria**						
Firmicutes	68.73	74.34	63.55	68.07	64.29	0.172
Patescibacteria	16.33	14.28	19.52	14.42	15.87	0.637
Cyanobacteria	5.45	2.13	5.75	6.37	4.24	0.189
Planctomycetota	1.14	0.32	0.74	0.64	1.08	0.158
Proteobacteria	1.87	0.97	2.80	2.72	2.85	0.114
Actinobacteriota	3.49	4.97	4.21	4.09	3.58	0.789
Bacteroidota	1.55	0.62	1.76	2.00	3.04	0.058
Verrucomicrobiota	1.14[B]	1.74[B]	1.16[B]	1.14[B]	3.83[A]	0.005
Euryarchaeota	0.07	0.08	0.02	0.24	0.11	0.356
Spirochaetota	0.02[b]	0.00[b]	0.02[b]	0.01[b]	0.07[a]	0.023
Total*	99.78	99.46	99.54	99.70	98.96	
**Fungus**						
Ascomycota	86.11	64.86	73.73	82.82	82.82	0.399
Basidiomycota	10.05	13.30	10.24	14.04	16.50	0.670
Chytridiomycota	0.59	0.32	0.20	0.21	0.10	0.528
Glomeromycota	0.34	0.07	0.02	0.07	0.08	0.213
Mortierellomycota	0.57	0.13	0.36	0.69	0.03	0.168
Mucoromycota	0.64	1.27	1.44	0.91	0.39	0.604
Neocallimastigomycota	0.65	19.90	13.76	0.78	0.01	0.419
Rozellomycota	0.57	0.05	0.03	0.27	0.04	0.109
Zoopagomycota	0.12	0.00	0.00	0.16	0.00	0.538
unclassified_Fungi	0.37	0.09	0.22	0.06	0.02	0.229
Total	100	100	100	100	100	

† In the same row, values with no superscript or the same letter superscript indicate no significant difference ($P > 0.05$). Values with different lowercase letter superscripts indicate a significant difference ($P < 0.05$), while values with different uppercase letter superscripts indicate a highly significant difference ($P < 0.01$). The same interpretation applies to the values below.

* The table shows the composition of the top 10 bacterial phyla.

Supplementary Table S4. Composition of duodenum bacteria and fungus at the genus level %

Composition*	Groups					*P*-value
	Con	PSMS5	PSMS10	PSMS15	PSMS20	
**Bacteria**						
Candidatus_Saccharimonas	16.2	14.27	19.51	14.41	15.84	0.636
uncultured_rumen_bacterium	9.36	11.64	11.36	10.17	13.49	0.738
Christensenellaceae_R_7_group	11.35	6.83	9.29	7.37	6.41	0.340
Lachnospiraceae_NK3A20_group	6.61	8.67	6.96	6.97	4.12	0.658
Mogibacterium	6.17	9.96	4.29	4.36	3.21	0.425
NK4A214_group	5.23	3.19	3.08	4.53	4.04	0.762
Family_XIII_AD3011_group	3.74	5.90	3.02	3.95	2.96	0.763
Ruminococcus	2.21	6.35	3.28	2.41	2.96	0.119
Bacillus	2.27	1.13	1.81	6.50	3.62	0.071
Lysinibacillus	2.38	0.85	1.19	5.00	2.18	0.075
Others	34.40	31.17	36.13	34.32	41.09	0.111
Unassigned	0.00	0.02	0.07	0.00	0.08	0.420
**Fungus**						
Aspergillus	28.53	33.68	32.97	22.34	38.95	0.817
Trichoderma	19.32	18.36	29.92	40.70	33.22	0.057
Phanerochaete	6.84	11.35	7.00	13.50	13.48	0.396
Orpinomyces	0.09	17.59	11.46	0.68	0.01	0.466
unidentified	11.21	1.49	1.73	7.09	0.71	0.138
Chaetomium	11.33	0.01	0.00	0.11	0.00	0.407
Thermoascus	2.09	1.66	1.15	0.46	0.65	0.409
Penicillium	0.70	0.88	1.14	1.87	0.92	0.591
Alternaria	0.72	0.37	1.41	0.26	1.76	0.487
Cladosporium	1.18	0.67	0.27	1.52	0.82	0.345
Others	18.00	13.94	12.94	11.47	9.50	0.217

* The table presents the composition of the top 10 genera of bacteria and fungi.

Supplementary Table S5. Effect of feeding different diets on predominant predicted gene pathways in the different ecological niches of growing Hu sheep

Items	Groups					*P*-value
	Con	PSMS5	PSMS10	PSMS15	PSMS20	
Microbial Picrust2 Prediction						
Global and overview maps	42.55	42.58	42.72	42.29	42.68	0.138
Carbohydrate metabolism	9.11	9.04	9.13	9.11	9.03	0.831
Amino acid metabolism	6.79[ab]	6.62[b]	6.81[ab]	7.00[a]	6.85[a]	0.022
Metabolism of cofactors and vitamins	4.36	4.33	4.27	4.29	4.31	0.853
Nucleotide metabolism	3.98	4.13	3.92	3.82	3.91	0.094
Translation	3.95	4.14	3.92	3.68	3.90	0.073
Energy metabolism	3.93	3.94	3.97	3.92	4.02	0.306
Membrane transport	3.80	3.76	3.77	3.98	3.73	0.116
Replication and repair	3.27	3.42	3.25	3.07	3.24	0.062
Signal transduction	2.48	2.38	2.45	2.63	2.46	0.150
Lipid metabolism	1.95	1.96	1.91	2.06	1.96	0.135
Folding, sorting, and degradation	1.60	1.62	1.59	1.54	1.61	0.144
Cellular community - prokaryotes	1.61[b]	1.57[b]	1.61[b]	1.7[a]	1.58[b]	0.024
Metabolism of other amino acids	1.27[b]	1.27[b]	1.27[b]	1.32[a]	1.31[ab]	0.037
Metabolism of terpenoids and polyketides	1.12	1.15	1.13	1.11	1.12	0.080
Glycan biosynthesis and metabolism	1.02	1.01	1.02	0.98	1.04	0.173
Drug resistance: Antimicrobial	0.86	0.87	0.86	0.87	0.86	0.917
Cell motility	1.13	1.02	1.17	1.19	1.05	0.287
Biosynthesis of other secondary metabolites	0.98	0.95	0.99	0.93	0.94	0.096
Xenobiotics biodegradation and metabolism	0.77[b]	0.71[b]	0.79[b]	0.94[a]	0.84[a]	0.034
Fungal Guild Prediction						
Wood Saprotroph	23.76	21.83	19.53	27.42	26.98	0.563
Plant-Pathogen	16.99	16.98	18.66	24.69	25.82	0.363
Endophyte	14.38	16.06	16.60	24.26	24.62	0.205
Undefined Saprotroph	22.67	13.34	14.67	12.11	11.35	0.229
Animal Endosymbiont	2.85	23.17	15.07	1.68	0.28	0.352
Animal Pathogen	12.96	4.23	11.14	5.15	9.15	0.114
Plant Saprotroph	1.77	2.92	2.40	0.21	0.08	0.273
Dung Saprotroph	1.50	1.09	0.16	3.90	0.06	0.167
Lichen Parasite	1.14	0.08	1.12	0.40	1.16	0.390
Ectomycorrhizal	0.88	0.00	0.08	0.02	0.20	0.377
Arbuscular Mycorrhizal	0.45	0.16	0.02	0.09	0.09	0.255
Epiphyte	0.00	0.00	0.40	0.02	0.03	0.465
Fungal Parasite	0.30	0.04	0.15	0.01	0.07	0.327
Lichenized	0.00	0.00	0.00	0.04	0.11	0.511
Soil Saprotroph	0.09	0.08	0.00	0.00	0.00	0.547
Plant Parasite	0.09	0.00	0.00	0.00	0.00	0.431
Ericoid Mycorrhizal	0.06	0.00	0.00	0.00	0.00	0.431
Litter Saprotroph	0.07	0.00	0.00	0.00	0.00	0.431
Leaf Saprotroph	0.03	0.00	0.00	0.00	0.00	0.431
Other	0.00	0.00	0.00	0.00	0.00	-
